# The Uptake and Translocation of Lead, Chromium, Cadmium, and Zinc by Tomato Plants Grown in Nutrient and Contaminated Nutrient Solutions: Implications for Food Safety

**DOI:** 10.3390/toxics13090738

**Published:** 2025-08-31

**Authors:** Radmila Milačič Ščančar, Katarina Kozlica, Stefan Marković, Pia Leban, Janja Vidmar, Ester Heath, Nina Kacjan Maršić, Špela Železnikar, Janez Ščančar

**Affiliations:** 1 Jožef Stefan Institute, Department of Environmental Sciences, Jamova cesta 39, 1000 Ljubljana, Slovenia; katarina.kozlica@ijs.si (K.K.); stefan.markovic@ijs.si (S.M.); pia.leban@ijs.si (P.L.); janja.vidmar@ijs.si (J.V.); ester.heath@ijs.si (E.H.); 2 Jožef Stefan International Postgraduate School, Jamova cesta 39, 1000 Ljubljana, Slovenia; 3 Biotechnical Faculty, Department of Agronomy, University of Ljubljana, Jamnikarjeva 101, 1000 Ljubljana, Slovenia; nina.kacjan.marsic@bf.uni-lj.si (N.K.M.); spela.zeleznikar@bf.uni-lj.si (Š.Ž.)

**Keywords:** lead, chromium, cadmium, zinc, uptake, nutrient solutions, tomato plants, translocation, food safety

## Abstract

The uptake and translocation of Pb, Cr, Cd, and Zn in tomato plants (*Solanum lycopersicum* L. Rally) were investigated. Tomato seedlings were grown for five weeks in pots containing 40 L of Hoagland nutrient solution (pH 7) or contaminated nutrient solutions at two concentration levels for each element: Cr (100 and 1000 ng/mL), Zn (100 and 1000 ng/mL), Pb (100 and 500 ng/mL), and Cd (50 and 500 ng/mL). The solutions were replenished weekly to maintain a volume of 40 L (pH 7), and 10 mL samples were collected for elemental analysis. After five weeks, the plants were harvested and separated into roots, stems, leaves, and fruits. These samples underwent microwave-assisted digestion, and the element concentrations were determined by inductively coupled plasma mass spectrometry (ICP-MS). The results revealed that the elements were mainly accumulated in the roots, with much lower concentrations determined in the fruits. Pb and Cr accumulated only minimally in fruits, with Pb levels of 0.0009 mg/kg wet weight at LI and 0.003 mg/kg wet weight at LII, and Cr levels of 0.028 mg/kg wet weight at LI and 0.031 mg/kg wet weight at LII. The Pb levels did not exceed the permissible limits set by EC regulations (0.05 mg/kg wet weight). Zn exhibited the highest accumulation in fruits, with 2.17 mg/kg wet weight at LI and 4.8 mg/kg wet weight at LII. By contrast, the Cd concentrations in fruits (0.25 mg/kg wet weight at LI and 1.1 mg/kg wet weight at LII) exceeded the EC regulatory limit of 0.02 mg/kg wet weight. The uptake of other essential elements into the tomato plant remained largely unaffected by the presence of contaminants. These results provide valuable insights into food safety. Laser ablation (LA)-ICP-MS imaging revealed an even distribution of Cd and Zn in the leaves of plants grown in contaminated nutrient solutions. By contrast, Cr and Pb were predominantly localized in the leaf veins and at the leaf apex, suggesting different transport mechanisms for these elements from the roots to the aerial parts of the plant.

## 1. Introduction

Growing concerns over the sustainability of freshwater use in agriculture have led to increased research into the use of river water, groundwater, well water, and lake water for irrigation [1,2,3,4,5,6,7]. Irrigation water is often contaminated by discharges from untreated industrial or mining sewage, household wastewater, and solid municipal waste disposal sites [1,3,6]. As another source of irrigation water, treated wastewater from municipal treatment plants is also being explored [2,8,9,10,11], and the quality of this wastewater is a decisive factor in determining its suitability for agricultural use [8]. Known contaminants often present in irrigation water include potentially toxic elements (PTEs) such as cadmium (Cd), lead (Pb), arsenic (As), chromium (Cr), and zinc (Zn) [1,6,10]. Cd, Pb, As, and hexavalent Cr are concerning due to their toxicity [12,13,14,15,16]. Although Cr is not an essential nutrient for plants, low levels of trivalent Cr may promote plant productivity, but high concentrations inhibit seed germination, reduce photosynthesis, and cause chlorosis and necrosis [17].

Zn is an essential micronutrient involved in plant growth and plays an important role in regulating nitrogen metabolism and photosynthesis [18]. While Zn is a key element in plant metabolism, high levels of ionic Zn inhibit photosynthesis and lead to toxicity [19]. By contrast, Cd and Pb are highly toxic elements; Cd induces oxidative stress and chromosomal aberrations, impairs photosynthesis, and reduces yield [20], while Pb inhibits plant growth, disrupts photosynthesis, induces chlorosis, and causes root system blackening due to oxidative stress and cellular damage [21].

Several international and national guidelines have been issued on acceptable concentrations of PTEs in irrigation water [22]. In general, the values established for drinking water can be considered a reference point for irrigation water. For example, the World Health Organization (WHO) recommends maximum allowable concentrations in drinking water of 0.003, 0.01, 0.01, and 0.05 mg/L for Cd, Pb, As, and Cr, respectively [23]. Although there are no specific WHO guidelines for Zn in drinking water, concentrations below 3 mg/L are generally considered safe [24]. Data from global research also show that PTE concentrations in irrigation water frequently exceed WHO guideline values, in some cases by an order of magnitude or more [1,2,3,5,6,7].

To predict possible health risks associated with irrigation water use, it is essential to understand the uptake and localization of contaminants in plants, particularly in the edible tissues [8]. Tomatoes are popular and one of the most widely consumed foods worldwide. They are known to be a rich source of nutrients and organic acids and contain several antioxidants, such as ascorbic acid, phenolic compounds, and flavonoids [25]. Depending on growth conditions, tomato fruits accumulate essential micronutrients as well as toxic compounds. Tomatoes have been frequently investigated for the presence of both essential and toxic elements [26], and numerous studies have focused on the uptake of PTEs from soil into the tomato plant. These investigations have included the distribution of PTEs in various parts of the plant, such as the roots, stems, leaves, and fruits [27,28,29], or solely the fruit, the only edible part of the tomato [30,31,32,33,34,35].

Studies have shown that the highest concentrations of PTEs are found in tomato roots, from where they are translocated to other parts of the plant, with the lowest concentrations in the tomato fruits [27,28]. In one study, as expected, the concentrations of PTEs in tomatoes grown in uncontaminated soil were very low and did not pose a health risk [29]. However, care should be taken if the soil, water, or wastewater used for irrigation is contaminated with PTEs [27,28,29,32,33,34]. In such cases, PTEs can be transferred to tomato fruits in concentrations that exceed the maximum permissible values established by relevant legislation. The European Union has set maximum permitted concentrations for tomato fruit of 0.02 mg/kg (wet weight) for Cd [36] and 0.05 mg/kg (wet weight) for Pb [37].

Hydroponic solutions are often used to understand the pathways of PTE uptake into plants, as they allow controlled experimental conditions and the contamination of nutrient solutions at different concentrations of selected contaminants [38,39,40]. Among the investigated metals, the uptake of toxic elements such as Cd and Pb into the tomato plant from hydroponic nutrient solutions has been frequently studied [41,42,43,44].

Plastic pots can adsorb PTEs from nutrient solutions, which reduces PTE concentrations in nutrient solutions. During ageing, plastics can fragment into micro- and nanoplastic particles (MPs and NPs), which may further increase adsorption rates in hydroponic or aqueous systems. Adsorption occurs through electrostatic interactions, complexation with polymer surface functional groups, and mechanical trapping within pores. The efficiency of these interactions depends on the type of plastic, particle size, surface area, solution pH, ionic strength, and PTEs properties such as charge, ionic radius, and speciation [45,46,47,48]. Although direct studies in hydroponic systems are limited, evidence from other aquatic environments suggests similar behavior.

Laser ablation (LA)-ICP-MS imaging is an effective analytical technique for visualizing the localization of elements absorbed by plants [49]. However, there are few studies using this method to investigate the distribution of elements within plant organs after exposure to contaminated nutrient solutions or irrigation with wastewater.

To contribute new knowledge on food safety, the objectives of the present study were (i) to investigate the uptake of Pb, Cr, Cd, and Zn in tomato plants (*Solanum lycopersicum* L. Rally) from control plants grown in uncontaminated nutrient solutions and plants grown in contaminated nutrient solutions at two concentration levels commonly found in irrigation water; (ii) to determine the concentrations of Pb, Cr, Cd, and Zn in the roots, stems, leaves, and fruits five weeks after plant exposure to both nutrient and contaminated nutrient solutions using ICP-MS; (iii) to evaluate the influence of contaminants added to the nutrient solution on the uptake of essential elements in plants, such as boron (B), sodium (Na), phosphorus (P), potassium (K), calcium (Ca), manganese (Mn), iron (Fe), copper (Cu), and molybdenum (Mo); (iv) to study the localization of Cd, Pb, Cr, and Zn in tomato leaves using laser ablation (LA)-ICP-MS imaging; and (v) to evaluate the potential health risks associated with the consumption of tomato fruits exposed to various concentration levels of toxic elements in contaminated nutrient solutions.

## 2. Materials and Methods

### 2.1. Instrumentation

Concentrations of elements were determined by ICP-MS using an Agilent 7900 instrument (Agilent Technologies Inc., Tokyo, Japan). For element mapping in tomato leaves, an Analyte G2 193 ArF excimer laser with a HelEx II low-dispersion ablation cell (Teledyne Photon Machines Inc., Bozeman, MT, USA) was coupled with an Agilent 8800 ICP-QQQ-MS (Agilent Technologies Inc., Tokyo, Japan) via ARIS (Aerosol Rapid Introduction System, Teledyne Photon Machines, Omaha, NE, USA). The HDIP LA imaging software (purchased from Teledyne Photon Machines, Belgrade, MT, USA) was used to create an elemental distribution map of the sample. The ICP-MS and LA-ICP-MS operating parameters are provided in the Supplementary Materials (Table S1). A CEM Corporation MARS 6 Microwave System (Matthews, NC, USA) was used to digest the plant samples. The pH was measured using a WTW pH meter 3110 (Weilheim, Germany). A Mettler AE 163 analytical balance (Mettler Toledo, Zürich, Switzerland) was used for weighing.

### 2.2. Reagents and Materials

Ultrapure (Milli-Q) water (18.2 MΩ × cm) was obtained from Direct-Q 5 (Millipore, Watertown, MA, USA) and was used in all the steps of sample preparation and analysis. Suprapur nitric acid (HNO_3_, 67–70%) obtained from Carlo Erba Reagents (Val-de-Reuil, France), and suprapur hydrofluoric acid (HF, 40%) and suprapur hydrogen peroxide (H_2_O_2_, 30% solution in water), both obtained from Merck (Darmstadt, Germany), were used in sample preparation and digestion. ICP multi-element standard solution XVI (21 elements in diluted nitric acid, 100 mg/mL) and stock standard solutions of scandium (Sc), germanium (Ge), yttrium (Y), rhodium (Rh), and indium (In) (1000 ± 2 mg/L in 2–3% HNO_3_) purchased from Merck were used to prepare calibration curves and internal standards for the determination of elements by ICP-MS. Hoagland nutrient solution (pH around 7) was prepared according to the procedure of Resh [50] using potable water and nutrients: 14.0 mM NO_3_^–^, 1.0 mM NH_4_^+^, 1.0 mM H_2_PO_4_^–^, 6.0 mM K^+^, 4.0 mM Ca^2+^, 2.0 mM SO_4_^2–^, 2.0 mM Mg^2+^, 10 μM Mn^2+^, 5.0 μM Zn^2+^, 30 μM BO_3_^3–^,15 μM Fe^3+^, 0.75 μM Cu^2+^, and 0.5 μM MoO_4_^2–^. Stock spike solutions of Cr, Zn, Cd, and Pb were prepared using Cr(NO_3_)_3_ × 9H_2_O, Zn(NO_3_)_2_ × 6H_2_O, Cd(NO_3_)_2_ × 4H_2_O, and Pb(NO_3_)_2_, all purchased from Merck. The certified standard reference material SPS-SW1 (Reference material for measurements of elements in surface waters), obtained from Spectrapure Standards (Oslo, Norway), was used to check the accuracy in the determination of elemental concentrations in nutrient solutions. SRM 1573a Tomato Leaves, purchased from NIST (National Institute of Standards and Technology, Gaithersburg, MD, USA), were used to check the accuracy of the determination of element concentration in plant samples. The stability of the LA-ICP-MS measurement was checked using SRM 612 Trace Elements in a glass standard reference material obtained from NIST. The 0.45 µm nitrocellulose filters (25 mm diameter), obtained from Sartorius, were used to prepare the calibration standards in the LA-ICP-MS procedure. SuperFrost Microscope Slides, used for fixing the samples and the calibration standards, were obtained from VWR (Radnor, PA, USA).

### 2.3. Experimental Conditions

Temperature (T) and relative humidity (RH) were recorded hourly in a greenhouse using a USB data logger (DL-121TH; Voltcraft, Hirschau, Germany). The daily mean air temperature (T_mean_) was calculated as the weighted average of the daily measured temperatures at 7 am, 2 pm, and 9 pm, with the 9 pm measurement given double weight: T_mean_ = (T_7_ + T_14_ + 2⋅T_21_)/4. The daily mean value of the relative humidity (RH_mean_) was calculated as the arithmetic mean of the relative humidity measured daily at 7 am, 2 pm, and 9 pm: RH_mean_ = (RH_7_ + RH_14_ + RH_21_)/3 [51]. These data are provided in Figure S1 of the Supplementary Materials, which shows the daily mean air temperature and daily mean relative humidity throughout the growth period.

The photosynthetically active radiation (PAR) in the greenhouse was continuously monitored using a Quantum Sensor LI-190R (LI-COR Biosciences, Lincoln, NE, USA) installed under the greenhouse roof. These measurements, combined with an average day length of 15 h during the growth period, were used to calculate the Daily Light Integral (DLI). The DLI varied considerably throughout the growth period, ranging from 13 to 23 mol/m^2^·d, which corresponds to PAR values of approximately 240 to 426 µmol/m^2^·s. These light conditions were favorable for the growth of tomato plants [52]. Two pronounced drops in DLI, to 3 and 5 mol/m^2^·d, were recorded during the second and fourth weeks after transplanting the plants into pots, respectively. The PPFD and DLI during the growth period are shown in Figure S2 of the Supplementary Materials.

### 2.4. Experimental Design

Seeds of *Solanum lycopersicum* L. Rally were sown in plug trays filled with peat substrate (Neuhaus N8). Once the seedlings developed five leaves (BBCH 105) and reached a height of 30 cm, the soil was carefully removed from the roots, the roots were washed with water, and the seedlings supplied by Terra Aquatica (Milan, Italy) were transferred to net pots from Aerofarm (Jersey City, NJ, USA), with three seedlings per plastic pot. Pots were placed in the greenhouse, each containing 40 L of either Hoagland’s nutrient solution (control, C) or a nutrient solution spiked with contaminants at two concentration levels: Level I (LI: 100 ng/mL of Pb, Cr, and Zn, and 50 ng/mL of Cd per pot) and Level II (LII: 1000 ng/mL of Cr and Zn, and 500 ng/mL of Pb and Cd per pot). The concentration levels of contaminants were similar to the literature data reported for moderate or highly polluted water used for irrigation [2,5] and for highly polluted irrigation water [2,6,7,9]. The selected contaminant levels were also aligned with the World Health Organization (WHO) Guidelines for the Safe Use of Wastewater in Agriculture [53], which recommend maximum concentrations of 5 mg/L for Pb, 0.1 mg/L for Cr, 0.01 mg/L for Cd, and 2 mg/L for Zn. In addition, the limits set by individual EU member states under Directive 91/271/EEC [54] were considered, which specify ranges of 0.5–5 mg/L for Pb, 0.01–0.1 mg/L for Cd, 0.1–1 mg/L for Cr, and 2–5 mg/L for Zn. The pots were covered with polystyrene foam lids to prevent water evaporation and algae growth. An air pump was used to aerate the nutrient solution.

During the experiment, samples (10 mL) of both nutrient and contaminated nutrient solutions were collected weekly for analysis of Pb, Cr, Cd, and Zn. The pH was determined in each pot. The nutrient solution or contaminated nutrient solutions lost due to evaporation or plant uptake were replenished to a total volume of 40 L, with the pH adjusted to 7 using HNO_3_. The contaminated nutrient solutions used for replenishment contained the same concentrations of contaminants (LI or LII) as those present at the start of the experiment. Plant growth and development were monitored weekly through visual assessments and phenological staging using the BBCH scale for tomato (*Solanum lycopersicum* L. Rally). Particular attention was paid to identifying visible nutrient or growth deficiencies. In such cases, the nutrient solution and environmental growth conditions were examined prior to further action. No intermediate destructive sampling was performed to minimize disturbance to plant growth. The experiment concluded at the stage of the first mature fruit (BBCH 809). The fresh mass of each plant component was measured, and samples were subsequently processed as described below. After five weeks of exposure, the plants were harvested and separated into roots, stems, leaves, and fruits. The fresh mass of each plant component was determined, and samples were subsequently processed as described below. Three leaves from each plant were selected for LA-ICP-MS analysis, and the remaining plant samples were cut into pieces using metallic iron scissors, dried at 60 °C, placed in an agate mortar, treated with liquid nitrogen, and ground with an agate pestle. The homogenized dried samples were decomposed using microwave-assisted digestion, and the moisture content was also determined.

**Figure 1:**
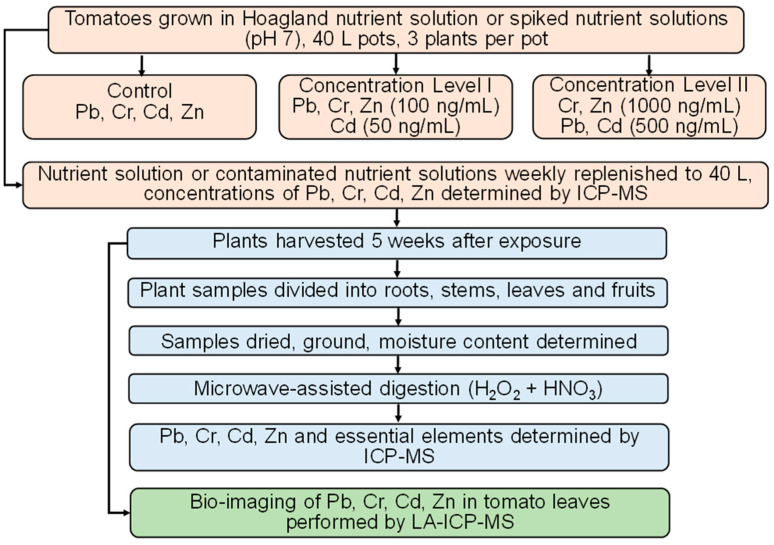
Flow chart showing the experimental design.

The impact of added contaminants (LI and LII) in the nutrient solution on the uptake of essential elements by the plants was also evaluated by measuring the concentrations of B, Na, P, K, Ca, Mn, Fe, Cu, and Mo in the digested samples. In these digested samples, element concentrations were determined by ICP-MS. The flow chart showing the experimental design is presented in Figure 1.

### 2.5. Analytical Procedures for the Determination of Elements in Nutrient Solutions and Plant Samples

Samples were acidified by adding 20 µL of HNO_3_ to 10 mL of the sample to determine the total concentrations of elements in nutrient solutions. For plants, approximately 0.2 g of homogenized dry sample was weighed into a Teflon vessel, to which 7 mL of H_2_O_2_, 1 mL of HNO_3_, and 0.05 mL of HF were added. The contents were subjected to microwave-assisted digestion using the following program: 20 min ramp to 140 °C, hold for 2 min, 15 min ramp to 200 °C, and hold for 60 min. After digestion, the obtained clear solutions were transferred into 20 mL graduated PE tubes and filled to the mark with MilliQ water. Concentrations of elements were determined by ICP-MS, using matrix-matched standards for calibration. To comply with Commission Regulations 2021/1323 and 2021/1317 [36,37], which require that analytical results for fruiting-like vegetables be expressed on a wet weight basis, the moisture content was determined by measuring weight loss at 60 °C. This approach allowed the results to be reported appropriately on a wet weight basis. All analyses were performed in duplicate.

### 2.6. Sample Preparation and Analysis for LA-ICP-MS Elemental Mapping

Tomato leaves were rinsed with water, allowed to dry for one hour, and then attached to a glass slide using double-sided Scotch tape, ensuring that the leaves were completely flat on the tape. The mounted samples were subsequently dried at room temperature for one week. Because of the large area of the tomato leaf, the measurement parameters for LA-ICP-MS imaging were adjusted to achieve a reasonable analysis time and adequate sensitivity. For this purpose, the laser beam used had an 80 µm square-shaped spot, providing uniform energy delivery with a fluence of 1.0 J/cm^2^. The system operated at a repetition rate of 100 Hz and a scan speed of 400 µm/s, with connected parallel lines for rastering. Each analysis cycle had a total dwell time of 0.2 s on the ICP-MS, resulting in a pixel size of 80 × 80 µm. The Aerosol Rapid Introduction System (ARIS) was employed for rapid analysis with effective signal retention. A helium carrier gas flow of 0.6 L/min was used to transport the ablated sample aerosol into the ICP, while 0.95 L/min of argon make-up gas was introduced via the ARIS mixing bulb. Under these operating conditions, the washout time for a single laser pulse was 60 ms. The LA-ICP-MS system was calibrated and tuned daily using the NIST SRM 612.

### 2.7. Calculation of the Translocation Factors

The translocation of Pb, Cr, Cd, and Zn from the roots to the aerial tissues of tomato plants was evaluated by calculating translocation factors (TFs) based on tissue dry weights and elemental concentrations, as outlined in Equations (1)–(3).

TF_root → stem_ = (Element concentration)_stem_/(Element concentration)_root_ (1)

TF_root → leaf_ = (Element concentration)_leaf_/(Element concentration)_root_ (2)

TF_root → fruit_ = (Element concentration)_fruit_/(Element concentration)_root_ (3)

### 2.8. Dietary Exposure and Health Risk Assessment

Dietary exposure to PTEs was assessed for both toddlers (children 1–3 years) and adults by combining data on Cd and Pb concentrations (wet weight) in tomatoes grown in contaminated nutrient solutions at concentration levels LI and LII, along with corresponding average- and high (97.5th percentile)-consumption data from the EFSA Comprehensive European Food Consumption Database [55]. The potential non-carcinogenic health risks associated with consuming tomatoes contaminated with Cd and Pb were evaluated using the hazard quotient (HQ) approach, as calculated in Equation (4):

HQ = DE/RfD(4)

where DE is the dietary exposure expressed in mg/kg bw per day, and RfD is the reference dose expressed in mg/kg bw per day. An HQ < 1 indicates the absence of a significant non-carcinogenic health risk, whereas an HQ > 1 denotes the possibility of adverse health effects. The DE was calculated according to Equation (5):

DE = Cm × Cd(5)

where Cm represents the concentration of the potentially toxic element, expressed in mg/g (wet weight), and Cd refers to the consumption of fresh tomatoes (average or high) in g/kg bw per day, with data varying at the individual level for toddlers and adults. Reference dose values for Cd and Pb used for calculating hazard quotients are presented in Table S2.

## 3. Results and Discussion

### 3.1. Accuracy Check, Limits of Detection, and Limits of Quantification

To verify the determination accuracy of elements by ICP-MS, SPS-SW1, certified for element concentrations in surface waters, was used. The determination accuracy of selected elements in plant samples was checked by analyzing SRM 1573a Tomato Leaves. For Pb determination, SRM 1573a was spiked with Pb, and a spike recovery test was performed. The results of the SPS-SW1, SRM 1573a, and spike recovery test are provided in Table S3. As is evident, good agreement between the determined elemental concentrations and the certified values was obtained (the differences did not exceed ±3%). The spike recovery test for Pb gave a 99% recovery. All these results confirmed the accurate determination of element concentrations in nutrient solutions and plant samples by ICP-MS. The limits of detection (LODs) and limits of quantification (LOQs) for the determination of elements by ICP-MS in nutrient solutions are presented in Table S4, whereas those in different parts of the tomato plant are shown in Table S5.

### 3.2. Concentrations of Pb, Cr, Cd, and Zn in the Nutrient and Contaminated Nutrient Solutions During the Experiment

The concentrations of Pb, Cr, Cd, and Zn in the contaminated nutrient solution were determined at the start of the experiment and during each weekly sampling throughout the experiment, before and after the replenishment of the contaminated nutrient solutions. In the control pot, concentrations were measured at the start and end of the experiment. The pH was also measured before and after each replenishment. The volume of nutrient solution was replenished to 40 L with nutrient or contaminated nutrient solutions at each weekly sampling. The contaminated nutrient solutions used for replenishment contained the same concentrations of contaminants (LI or LII) as those present at the start of the experiment. The results are provided in Table 1.

The volume of nutrient solution used by the plant for growth, or lost due to evaporation, steadily increased from 2 L after 7 days to 25 L throughout the experiment. The root exudates raised the pH from approximately 7 to around 8, which is consistent with previous observations of root-induced alkalinization in hydroponic systems [56]. Therefore, the pH was adjusted back to 7 by adding an appropriate amount of HNO_3_ during replenishments. It is evident that with each subsequent sampling, the concentration of the added contaminants (LI or LII) significantly decreased, either due to the uptake of metals by the tomato plant or the precipitation of Pb and Cr hydroxides and the adsorption of Cd^2+^ and Zn^2+^ cations onto the walls of the container. The adsorption phenomena on the plastic surfaces that reduce PTEs concentrations in nutrient solutions have also been reported by other researchers in similar studies conducted in aquatic environments [45,46,47,48]. After replenishment, the concentrations in the pot with contaminated nutrient solution slightly increased, with the increase being more pronounced at larger volumes of added contaminated solution.

### 3.3. Uptake of Pb, Cr, Cd, and Zn in Tomato Plant from Nutrient and Contaminated Nutrient Solutions

**Figure 2:**
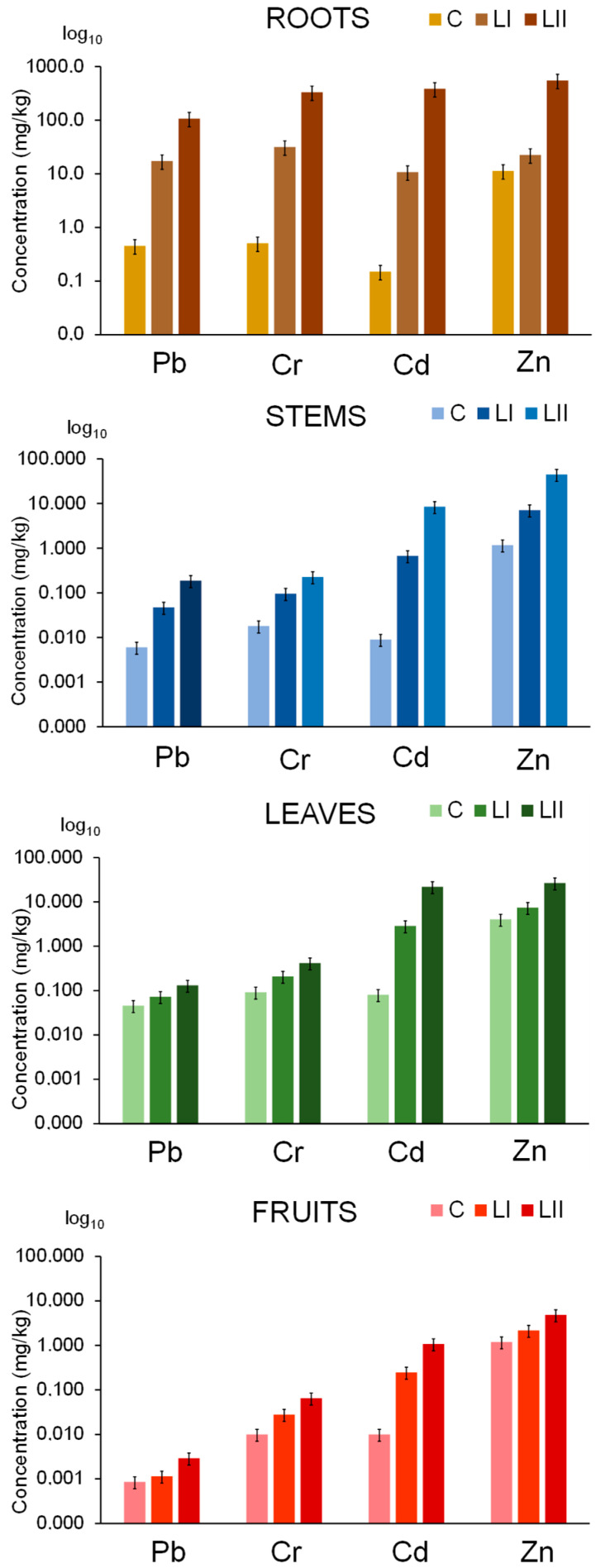
The uptake of Pb, Cr, Cd, and Zn in the tomato plant from the nutrient solution (C) and nutrient solution contaminated with Pb, Cr, Cd, and Zn at concentration levels LI and LII. The height of the bars represents elemental concentrations expressed on a wet weight basis as an average of three plants, with the error bars indicating the relative standard deviation (%).

The uptake of Pb, Cr, Cd, and Zn in the tomato plant from both nutrient and contaminated nutrient solutions was studied five weeks after exposure, in the stage of the first mature fruits (BBCH 809), when they began to turn reddish. The experiment was terminated at this point due to disease onset, which caused some fruits to begin decaying, ensuring that only healthy plant tissues were sampled. The plants were harvested, and the concentrations of elements in the digested samples of roots, stems, leaves, and fruits were determined by ICP-MS. The results of these experiments are shown in Figure 2. Elemental concentrations in the digested samples, corresponding to these results, are given in the Supplementary Materials (Table S6).

The results show that Pb, Cr, Cd, and Zn were predominantly accumulated in the roots of both control plants and those grown in contaminated nutrient solutions. For example, in plants exposed to a nutrient solution at concentration level LII, tomato roots accumulated the highest concentrations of these elements—107 mg/kg of Pb, 332 mg/kg of Cr, 384 mg/kg of Cd, and 551 mg/kg of Zn—whereas control plants contained only 0.454 mg/kg of Pb, 0.508 mg/kg of Cr, 0.150 mg/kg of Cd, and 11.3 mg/kg of Zn. In the upper parts of the plant, concentrations of Pb, Cr, Cd, and Zn were highest in the leaves and lowest in the tomato fruit. These findings are consistent with studies of tomatoes irrigated with wastewater [27,28,29].

To assess the significance of differences between control plants and plants grown in contaminated nutrient solutions at concentration levels LI and LII, One-Way ANOVA was conducted. The results for Pb, Cr, Cd, and Zn in the roots, stems, leaves, and fruits of tomato plants are summarized in Table S7 of the Supplementary Materials. The analysis revealed statistically significant differences in metal accumulation between control plants and those exposed to contaminated nutrient solutions (*p* < 0.05, *F* > *F critical*). The results from ANOVA provide evidence that exposure to contaminated nutrient solutions has a significant impact on metal accumulation in different plant tissues.

Translocation from the roots to the aerial parts of the plant occurs through various pathways. Pb and trivalent Cr are not readily available at physiological pH values, forming poorly soluble hydroxides, such as Pb(OH)_2_ and Cr(OH)_3_, that plants cannot easily absorb [57,58]. However, low-molecular-mass (LMM) organic acids, such as malic, citric, and oxalic acids, released by root cells, can solubilize these hydroxides and subsequently form negatively charged complexes with Pb and Cr, facilitating their transport through root cell membranes, primarily via the symplastic pathway [59,60].

Once Pb and Cr are solubilized, the apoplastic pathway also plays a role in their transport. After being taken up by the roots, Pb transport to the rest of the plant is limited due to its binding with phytochelatins. The translocation of Pb is further reduced because it is sequestered in root vacuoles, thereby restricting its movement to other parts of the plant. Within these vacuoles, Pb undergoes complexation with glutathione and amino acids like proline [61]. Its immobilization also occurs through the formation of insoluble Pb phosphate [60,62]. These processes jointly contribute to the poor transfer of Pb from the roots to the fruit.

The transport of Cr from the roots to the upper parts of the plant is slightly more pronounced, as Cr-phosphate compounds are more soluble than those of Pb. However, other processes in the root vacuoles, such as Cr(III) sequestration or the binding to cell wall components, also inhibit Cr transport. The majority of Cr translocated from the roots is likely present as Cr(III)–LMM complexes. This is consistent with our previous findings on Cr(III)–LMM speciation in dandelion roots and leaves [49] and is supported by studies highlighting the role of LMM organic acids in metal homeostasis [59] and the mechanisms of uptake, transport, and accumulation in hyperaccumulator plants [63].

Cd and Zn are present as positively charged ions (Cd^2+^ and Zn^2+^) in the nutrient solution at pH 7 and follow similar transport mechanisms. They readily form complexes with LMM organic acids and are transported to the roots via the symplastic pathway or directly through calcium channels or apoplastic routes. The transport of Cd and Zn is also mediated by metal chelators, such as phytosiderophores and metallothioneins [59,60,63]. In comparison to Pb and Cr, Cd and Zn transport to the upper parts of the plant is more efficient.

Previous studies on hydroponic solutions have primarily focused on the toxic effects of Pb [44] and Cd [43], exposing tomato plants to metal concentrations at least ten times higher than those used in our experiments at concentration level LII (500 ng/mL). Consequently, the levels of Pb and Cd that accumulated in the fruits are not directly comparable to our results. However, reports on Cd and Zn accumulation in tomato fruits from plants grown in soil irrigated with moderately or highly polluted wastewater [32,33,34] reveal similar patterns of substantial accumulation, as observed in our study. While direct comparisons are difficult due to differences in experimental conditions, our findings suggest that Cd and Zn can be significantly transferred into tomato fruits when contaminated water is used for irrigation.

### 3.4. Translocation of Pb, Cr, Cd, and Zn from the Roots to the Aerial Parts of the Tomato Plant

To gain better insight into the translocation of Pb, Cr, Cd, and Zn from the roots to the aerial parts of the tomato plant, translocation factors (TFs) were calculated and are presented in Table 2.

Data from Table 2 indicate that Pb has the lowest uptake from the roots to the aerial parts of the plant, whereas Cr shows a slightly higher uptake. TFs below 0.15 indicate that Pb and Cr are largely retained in the roots, supporting the hypothesis that these metals are sequestered in root vacuoles, which limits their transport to the aerial parts of the plant [59]. Furthermore, data in Table 2 show that the translocation of Pb and Cr to the aerial parts of the plant is highest in the control plants, and decreases significantly as the concentrations of contaminants in the nutrient solutions increase at LI and LII. In control tomato plants, the TF from the roots to leaves was 0.326 for Pb and 0.398 for Cr. By contrast, plants exposed to Pb and Cr at concentration level LII exhibited a sharp decline in TFs, dropping to 0.0042 for Pb and 0.0017 for Cr. This reduction likely reflects sequestration in root vacuoles, which act as a detoxification mechanism by immobilizing Pb and Cr. As a result, their transport to stems and other aerial parts is restricted, protecting the plant from Pb toxicity and limiting the accumulation of the non-essential element Cr. Similarly, the TF from roots to fruits in control plants was 0.014 for Pb and 0.145 for Cr, whereas in contaminated plants, TFs were markedly lower: 0.0004 and 0.0002 for Pb at LI and LII, respectively, and 0.086 and 0.0014 for Cr. Correspondingly, Pb concentrations in tomato fruits were very low, ranging from 0.0006 mg/kg in control plants to 0.0009 and 0.0030 mg/kg in fruits of plants exposed to LI and LII contamination levels, respectively (Figure 2, Table S6). Cr concentrations in fruits increased from 0.01 mg/kg in the controls to 0.028 mg/kg and 0.31 mg/kg in the fruits of plants exposed to LI and LII, respectively. These concentrations do not pose a health risk, as the tolerable upper intake level for Cr(III) in adults is 1 mg/day [64].

Compared to Pb and Cr, significantly higher amounts of Cd and Zn are translocated from the roots to the aerial parts of both the control plants and those exposed to contaminated nutrient solutions at concentration levels LI and LII (Figure 2, Table 2). The transport of Cd and Zn from the roots is primarily mediated by phytosiderophores and metallothioneins, which form strong complexes with Cd^2+^ and Zn^2+^, as well as by LMM organic acids that form moderately stable complexes [59,63], facilitating the efficient transfer of these metals to the fruits. At the same level of nutrient solution contamination (LII: 500 ng/mL of Cd and Pb, and 1000 ng/mL of Cr and Zn) (Table S6), the Cd concentration in the fruits was 360 times higher than that of Pb, while the Zn concentration was 150 times higher than that of Cr. Data in Table 2 further show that in the control tomato plant, TFs from the roots to stems are 0.355 for Cd and 0.45 for Zn, while TFs from the roots to leaves are much higher, 1.81 and 0.738, respectively. TFs from the roots to fruits are also appreciably high, 0.738 for Cd and 1.04 for Zn. With increasing contamination levels, TFs for Cd decrease at both LI and LII, while for Zn, a decrease was observed at LII. The TFs from the roots to fruits at LII were 0.02 for Cd and 0.063 for Zn. This decline is associated with the synthesis of phytochelatins in response to metal stress, which helps mitigate damage from elevated Cd and Zn concentrations [63]. According to data in Figure 2 and Table S6, the fruits of control plants contained 0.01 mg/kg of Cd and 1.2 mg/kg of Zn. Despite plant defense mechanisms against metal stress, fruits from plants grown in contaminated nutrient solutions accumulated 0.25 mg/kg of Cd and 2.2 mg/kg of Zn at LI, and 1.1 mg/kg of Cd and 4.8 mg/kg of Zn at LII. The highest concentrations of Zn in tomato fruits (at LI and LII) do not pose a health risk, as Zn is an essential nutrient and the tolerable upper intake level for adults is 40 mg/day [65]. By contrast, Cd, classified as a human carcinogen, may pose health risks, especially for plants growing in contaminated environments [66].

To protect human health, the European Union has established maximum permissible concentrations for fruiting-like vegetables: 0.02 mg/kg (wet weight) for Cd [36] and 0.05 mg/kg (wet weight) for Pb [37]. In tomato fruits from plants exposed to LI and LII contamination, Pb concentrations were over 15 times below the maximum allowable limits, posing no health risk. However, Cd concentrations in these fruits exceeded the permissible levels by 12 times at LI and 50 times at LII.

Literature data on the translocation of elements from the roots to the aerial parts of tomatoes are mostly based on studies conducted on plants grown in soil irrigated with contaminated wastewater. Therefore, a direct comparison of TFs of elements from the roots to the aerial parts of the plant is difficult, as the translocation rate strongly depends on the chemical properties of the individual element and the concentration of contaminants in the growth medium. Nevertheless, some similarities with our work can be observed in the study by Tiwari et al. [67] on the translocation of elements in tomatoes grown in soil irrigated with contaminated industrial effluent. Consistent with our findings, they observed that Pb and Cr have low mobility from the roots, which act as a barrier limiting their translocation to the aerial parts of the plant. They reported that the TFs for Pb and Cr were approximately 0.15 from the roots to stems, around 0.05 from the roots to leaves, and the lowest, about 0.01, from the roots to fruits. By contrast, Cd and Zn were much more mobile and were substantially translocated to the aerial parts, in line with our observations. The TFs from the roots to stems and roots to leaves were approximately 0.5 for Cd and 1.1 for Zn, respectively, while those from the roots to fruits were around 0.1 for Cd and 0.6 for Zn.

### 3.5. Health Risk Assessment

To assess the non-carcinogenic health risks associated with consuming tomatoes contaminated with toxic elements like Pb and Cd, the hazard quotient (HQ) was calculated (see Section 2.8. *Dietary exposure and health risk assessment*). This task involved comparing Cd and Pb exposure levels against established safety thresholds like the reference dose (RfD), which defines the maximum daily intake considered safe over a lifetime for humans. The estimated daily intake and HQ for toddler and adult population groups are provided in Table S8.

For Pb, HQ values for toddlers were 0.0002 and 0.0012 at concentration levels LI and LII, respectively, and for adults, they were 0.0001 and 0.0005 for average consumption. At higher consumption levels, the values for toddlers were 0.0006 and 0.0039 at LI and LII, respectively, and for adults, they were 0.0003 and 0.0016. All HQ values remained well below 1, indicating that there is no potential health risk from Pb by consuming these tomatoes.

By contrast, the HQ values for Cd were higher. For average consumption, the values for toddlers were 0.53 at concentration level LI and 3.22 at LII, while for adults, they were 0.27 at LI and 1.32 at LII. For higher consumption levels, the values for toddlers increased to 2.30 at LI and 14.0 at LII, whereas for adults, they were 1.17 at LI and 5.76 at LII. At average consumption and LI, HQ values remained below 1 for both toddlers and adults. However, at LII, they exceeded 1. With high tomato consumption, HQ values greatly surpassed 1 for both toddlers and adults at both concentration levels, raising particular concern for toddlers at the LII contamination level with Cd. This finding suggests a potential health risk when tomatoes are cultivated using irrigation water polluted with Cd. Consequently, such tomatoes should not be consumed, particularly by toddlers. It is essential to monitor and regulate the levels of toxic elements in irrigation water through appropriate treatment, with particular attention given to Cd, as it readily accumulates in fruits.

### 3.6. The Influence of Added Pb, Cr, Cd, and Zn Contaminants in the Nutrient Solution on the Uptake of Essential Elements in Tomato Plants

**Figure 3:**
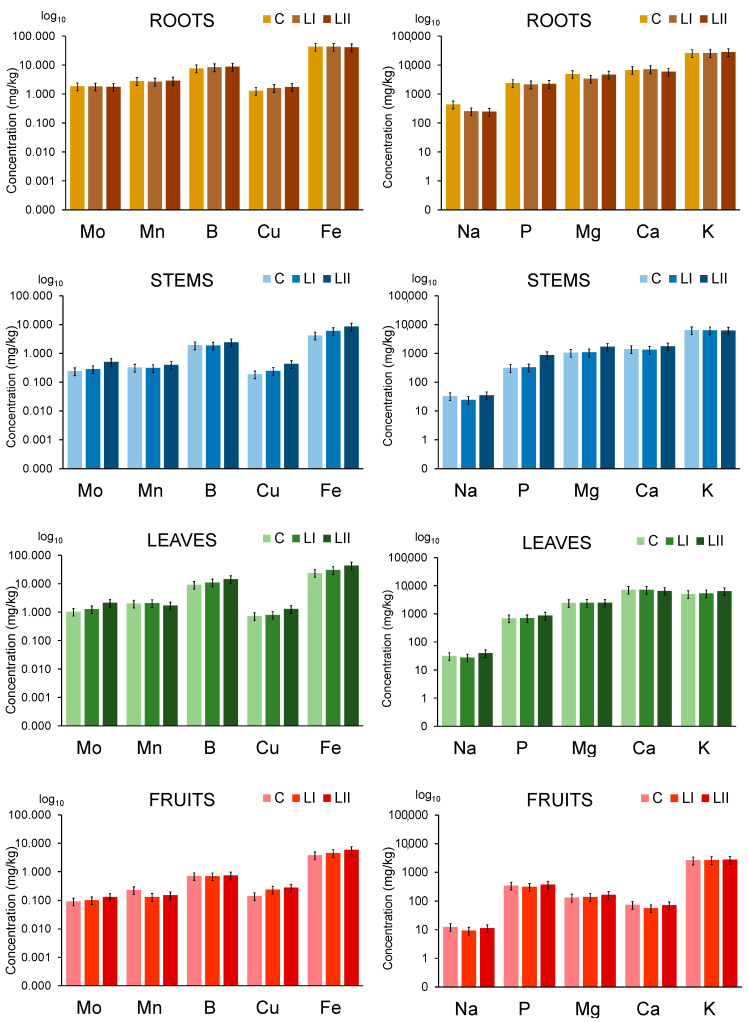
The uptake of essential elements in the tomato plant from nutrient solutions (C) and nutrient solutions contaminated with Pb, Cr, Cd, and Zn at concentration levels LI and LII. The height of the bars represents element concentrations expressed on a wet weight basis as an average of three plants, with the error bars indicating the relative standard deviation (%).

To evaluate the influence of contaminants added to the nutrient solution on the uptake of essential elements in plants, their concentrations were determined in different plant parts. The results are presented in Figure 3, while the elemental concentrations related to data from Figure 3 are provided in the Supplementary Materials (Table S6).

The presence of contaminants in the nutrient solution at concentration levels LI and LII does not significantly influence the uptake of micro-essential elements (Mo, Mn, B, Cu, and Fe) and macronutrients (Na, P, Mg, Ca, and K) in the upper parts of the tomato plant, as their concentrations do not differ significantly compared to control plants.

### 3.7. Imaging of Pb, Cr, Cd, and Zn in Tomato Leaves by LA-ICP-MS

**Figure 4:**
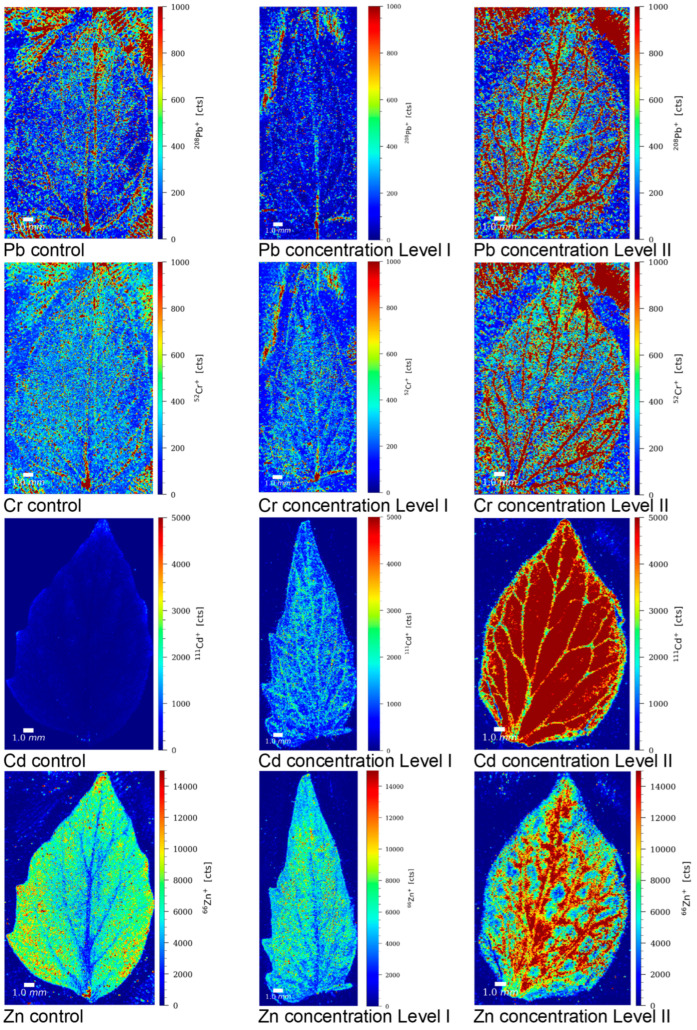
LA-ICP-MS images of tomato leaves showing the relative distribution of Pb, Cr, Cd, and Zn in the control sample (C) and samples grown in nutrient solutions contaminated with Pb, Cr, Cd, and Zn at concentration levels LI and LII. The color scale (red to blue; highest to lowest) denotes the elemental concentrations.

In order to gain a better understanding of the transport mechanism of Pb, Cr, Cd, and Zn from the roots to the upper parts of the tomato plant, the localization of these elements was examined in control tomato leaves and leaves from plants grown in contaminated nutrient solutions at concentration levels LI and LII. Element mapping was conducted using LA-ICP-MS, following the procedure described in Section 2.6*. Sample preparation and analysis for LA-ICP-MS elemental mapping.* The LA-ICP-MS images of tomato leaves showing the relative distribution of Pb, Cr, Cd, and Zn in the control sample and samples grown in contaminated nutrient solutions are presented in Figure 4.

The results reveal that Cd and Zn were evenly distributed in the tomato leaves of plants grown in contaminated nutrient solutions. By contrast, Cr and Pb were predominantly localized in the leaf veins, the vascular tissues responsible for transporting water and nutrients into the leaf. Additionally, they were also accumulated at the leaf apex. These observations suggest that the transport of Cd and Zn from the roots is more efficient, as it is facilitated by metal chelators like phytosiderophores and metallothioneins [59,63]. Conversely, the transfer of Pb and Cr from the roots to the upper parts of the plant is restricted due to the sequestration of these elements in root vacuoles, Pb binding with phytochelatins, and Cr(III) binding to the cell wall components [49,59,63]. These inhibitory effects and differing transport mechanisms result in distinct patterns of Pb and Cr localization in tomato leaves compared to Cd and Zn. The elemental mapping in leaves provided valuable insights into the transport mechanisms and further supported our findings on the accumulation and localization of elements in both control tomato plants and those grown in contaminated nutrient solutions.

## 4. Conclusions

Our investigation provides novel insights into the uptake and translocation of toxic elements Pb and Cd, the non-essential trivalent Cr, and the essential element Zn from moderately and highly contaminated Hoagland nutrient solutions to tomato plants. The results revealed that these elements were predominantly accumulated in the tomato roots. Translocation from the roots to the upper parts of the plant was most pronounced for Cd and Zn, while it was lower for Pb and Cr. Pb concentrations in tomato fruits remained well below the permissible levels set by EC regulations, whereas Cd exceeded the legislative limits by 12 and 50 times at contamination levels LI and LII, respectively. The health risk of consuming these tomatoes due to Pb contamination was low (HQ < 1). However, the HQ values for Cd exceeded 1 for both toddlers and adults at the average consumption rate at LII. At higher consumption rates, HQ values greatly exceeded 1 under both LI and LII for toddlers and adults. This important and novel finding indicates a potential health risk associated with growing tomatoes using irrigation water heavily contaminated with Cd. Therefore, such tomatoes should not be consumed, particularly by toddlers. Care should be taken when applying wastewater for irrigation, especially concerning toxic elements such as Cd, which can readily accumulate in tomato fruits. Our study also provides new evidence that the presence of contaminants in the nutrient solution does not markedly alter the uptake of other essential elements by the tomato plant. The LA-ICP-MS imaging of tomato leaves from plants grown in contaminated nutrient solution showed an even distribution of Cd and Zn, whereas Cr and Pb were primarily localized in the leaf veins and at the leaf apex. The different localization of the studied elements was attributed to distinct transport mechanisms from the roots to the upper parts of the tomato plant. Our research makes a significant contribution to advancing food safety knowledge. Understanding the uptake and translocation of toxic and essential elements from nutrient solutions to tomato plants is crucial for the accurate assessment and maintenance of food safety. In this context, a future challenge in our research is to use isotopically enriched elements to distinguish between the naturally occurring concentration of an element in Hoagland nutrient solution, which is taken up during plant growth, and the concentration of its enriched isotope taken up by the tomato plant after its intentional addition to the solution. This innovative approach will provide deeper insights into the mechanisms of element uptake and translocation in tomato plants and will enhance the reliability of analytical data used for food safety assessment.

## Figures and Tables

**Table 1:** Concentrations of Pb, Cr, Cd, and Zn in the nutrient solution (C = control) or contaminated nutrient solutions (LI = concentration level I, and LII = concentration level II) monitored throughout the experiment, both before (a) and after (b) replenishment. Element concentrations were determined using ICP-MS. The results represent mean concentrations obtained from two parallel samples, with a measurement uncertainty of better than ±3%.

**1st sampling at the start of the experiment**						
Samples	Pb (ng/mL)	Cr (ng/mL)	Cd (ng/mL)	Zn (ng/mL)	pH	The volume (L) of nutrient solution or contaminated nutrient solutions added at the start of the experiment
C-1a	0.622	0.519	0.283	20.7	7.1	40
LI-1a	90.7	107	54.0	125	6.7	40
LII-1a	541	1166	591	1144	6.7	40
**2nd sampling after 7 days**						
Samples	Pb (ng/mL)	Cr (ng/mL)	Cd (ng/mL)	Zn (ng/mL)	pH	The volume (L) of nutrient solution or contaminated nutrient solutions replenished to 40 L
C-2a	/	/	/	/	8.0	2.0
C-2b	/	/	/	/	7.0	2.0
LI- 2a	1.25	0.594	32.7	50.4	7.5	2.0
LI-2b	6.2	6.11	34.6	56.2	7.3	2.0
LII- 2a	0.71	0.72	74.9	71.3	7.5	2.0
LII-2b	21.7	53.3	104	130	7.3	2.0
**3rd sampling after 14 days**						
Samples	Pb (ng/mL)	Cr (ng/mL)	Cd (ng/mL)	Zn (ng/mL)	pH	The volume (L) of nutrient solution or contaminated nutrient solutions replenished to 40 L
C-3a	/	/	/	/	7.7	10
C-3b	/	/	/	/	7.0	10
LI-3a	2.14	0.705	35.7	59.7	7.3	10
LI-3b	27.4	32.7	40.7	72.4	6.8	10
LII-3a	2.10	1.19	82.4	105	7.2	10
LII-3b	101	301	192	298	6.7	10
**4th sampling after 21 days**						
Samples	Pb (ng/mL)	Cr (ng/mL)	Cd (ng/mL)	Zn (ng/mL)	pH	The volume (L) of nutrient solution or contaminated nutrient solutions replenished to 40 L
C-4a	/	/	/	/	7.4	15
C-4b	/	/	/	/	7.1	15
LI-4a	12.4	0.896	41.6	75.0	7.3	15
LI-4b	36.9	47.1	43.0	83.7	6.6	15
LII-4a	17.6	12.3	119	151	7.1	15
LII-4b	88.7	320	233	315	6.8	15
**5th sampling after 28 days**						
Samples	Pb (ng/mL)	Cr (ng/mL)	Cd (ng/mL)	Zn (ng/mL)	pH	The volume (L) of nutrient solution or contaminated nutrient solutions at the end of the experiment
C-5a	/	/	/	/	7.5	25
C-5b	/	/	/	/	6.7	25
LI- 5a	47.3	6.5	119	339	7.9	25
LI- 5b	59.5	45.1	61.0	161	6.9	25
LII-5a	64.3	34.3	262	547	8.1	25
LII-5b	207	324	351	839	6.9	25
**6th sampling after 35 days at the end of the experiment**						
Samples	Pb (ng/mL)	Cr (ng/mL)	Cd (ng/mL)	Zn (ng/mL)	pH	The volume (L) of nutrient solution or contaminated nutrient solutions at the end of the experiment
C-6a	0.413	0.191	1.23	48.7	7.4	17
LI- 6a	35.4	4.60	78.2	159	7.7	17
LII-6a	42.6	32.4	178	541	7.8	17

**Table 2:** Translocation factors (TFs) of Pb, Cr, Cd, and Zn from the roots to the aerial parts of the tomato plant in the control sample (C) and samples grown in contaminated nutrient solutions at concentration levels LI and LII.

Concentration Level	Pb		
	TF_root → stem_	TF_root → leaf_	TF_root → fruit_
C	0.083	0.326	0.014
LI	0.010	0.010	0.0004
LII	0.0042	0.0023	0.0002
	Cr		
C	0.141	0.398	0.145
LI	0.0081	0.016	0.0086
LII	0.0017	0.0023	0.0014
	Cd		
C	0.355	1.81	0.738
LI	0.257	0.691	0.217
LII	0.054	0.109	0.020
	Zn		
C	0.450	0.932	1.04
LI	1.34	0.850	0.938
LII	0.198	0.092	0.063

C = control, LI = concentration level I, LII = concentration level II.

## Data Availability

Data will be made available upon request to the corresponding authors.

## Supplementary Materials

The following supporting information can be downloaded at https://www.mdpi.com/article/10.3390/toxics13090738/s1. Table S1. ICP-MS and LA operating parameters. Table S2. Reference dose values for Cd and Pb used for calculating hazard quotients [51]. Table S3. Concentrations of elements in the standard reference material SPS-SW1 (Reference material for measurements of elements in surface waters) and certified reference materials CRM 1573a (Tomato leaves). Concentrations of elements were determined by ICP-MS. The results represent the mean concentration obtained on six parallel samples. Table S4. LODs and LOQs for the determination of the elements in the nutrient solution. LODs and LOQs were calculated as the concentration providing a signal equal to 3 *s* or 10 *s* of the blank sample, respectively. To calculate the LODs and LOQs, 8 blank samples were analyzed by ICP-MS. Table S5. LODs and LOQs for the determination of the elements in different parts of the tomato plant (wet weight basis). LODs and LOQs were calculated as the concentration providing a signal equal to 3 *s* or 10 *s* of the blank sample, respectively. To calculate the LODs and LOQs, 8 blank samples were analyzed by ICP-MS. Table S6. The uptake of contaminants (Pb, Cr, Cd, and Zn) and essential elements in the presence of contaminants at concentration levels LI and LII in different parts of the tomato. Element concentrations, determined by ICP-MS after the microwave-assisted digestion of samples, represent the average of three parallel samples and are expressed on a wet weight basis. The measurement uncertainty of ICP-MS is better than ±3, while the relative deviation in concentration between three parallel samples does not exceed ±30%. Table S7. One-Way ANOVA of Pb, Cr, Cd, and Zn concentrations in the tomato roots, stems, leaves, and fruits of control plants and plants grown in contaminated nutrient solutions at concentration levels LI and LII, with *p*-values indicating the level of significance (*p* < 0.05). Table S8. Estimated daily intake and hazard quotient for toddler and adult population groups. Figure S1. Daily mean air temperature (T_mean_) and relative humidity (RH) during the plant growth period in Ljubljana, 2022. Tmean ranged from 13 to 28 °C, with recorded extremes of 10 °C (T_min_) and 40 °C (T_max_). Figure S2. Photosynthetic Photon Flux Density (PPFD, primary axis) and Daily Light Integral (DLI, secondary axis) during the growth period in Ljubljana, 2022.
