# Supplementary material for: The Uptake and Translocation of Lead, Chromium, Cadmium, and Zinc by Tomato Plants Grown in Nutrient and Contaminated Nutrient Solutions: Implications for Food Safety
Source: Toxics. 2025 Aug 31;13(9):738. doi: 10.3390/toxics13090738 (PMC12474179; doi:10.3390/toxics13090738)
Supplement: Supplementary file 1 [file toxics-13-00738-s001.zip › toxics-3806832-supplementary.pdf]

Supplementary Materials

Table S1. ICP-MS and LA operating parameters.

ICP-MS operating parameters

Method	ICP-MS	LA-ICP-MS
ICP-MS instrument	Agilent 7900	Agilent 8800
Parameter	Type/Value	Type/Value
Aerosol introduction	Miramist	ARIS
Spray chamber	Scott	/
Skimmer and sampler	Ni	Ni
Plasma conditions		
Forward power	1550 W	1550 W
Plasma gas flow (Ar)	15.0 L/min	15.0 L/min
Carrier gas flow (He)	/	0.60 L/min
Carrier gas flow (Ar)	1.10 L/min	0.95 L/min
Makeup gas flow (Ar)	0.10 L/min	/
He gas flow	4.5 mL/min	/
Total carrier gas flow	1.20 L/min	1.55 L/min
Sample-uptake rate	0.3 mL/min	/
Data-acquisition parameters		
m/z of isotopes monitored	^{11}B, ^{23}Na, ^{24}Mg, ^{31}P, ^{39}K, ^{44}Ca, ^{52}Cr, ^{55}Mn, ^{56}Fe, ^{63}Cu, ^{66}Zn, ^{95}Mo, ^{111}Cd, ^{208}Pb	^{52}Cr, ^{66}Zn, ^{111}Cd, ^{208}Pb
m/z of internal standard	^{72}Ge, ^{103}Rh, ^{193}Ir	/

Laser operating parameters

Instrument	Analyte G2
Parameter	Type/Value
Wavelength	193 nm
Sample chamber	HelEx-II
Laser energy (Fluence)	1.0 J/cm
Laser- beam size	80 µm × 80 µm (square)
Scanning speed	400 µm/s
Repetition rate	100 Hz
Carrier gas flow (He)	0.60 L/min

Table S2. Reference dose values for Cd and Pb used for calculating hazard quotients [55].

Element	Reference value	Reference type*	Population	Year
Cd	2.5 µg/kg bw/week	TWI	Consumers	2011
Pb	25.0 µg/kg bw/week	TWI	Consumers	2005

*TWI – tolerable weekly intake

Table S3. Concentrations of elements in the standard reference material SPS-SW1 (Reference material for measurements of elements in surface waters) and certified reference materials CRM 1573a (Tomato leaves). Concentrations of elements were determined by ICP-MS. The results represent the mean concentration obtained on six parallel samples.

Element	SPS-SW1		CRM 1573a	
	Determined (µg/L)	Certified (µg/L)	Determined (mg/kg)	Certified (mg/kg)
Cd	0.51±0.02	0.50±0.01	1.48±0.04	1.517±0.027
Cr	1.99±0.03	2.00±0.02	2.02±0.06	1.988±0.034
Pb	4.89±0.08	5.0±0.1	/	/
Zn	19.7±0.4	20[a]	30.8±0.9	30.94±0.55
B	51.2±0.6	50[a]	33.20±0.97	33.13±0.42
Na	2010±10	2000±20	137±5	136.1±3.7
Mg	398±4	400±4	/	/
P	98.8±0.9	100±1	2170±40	2161±28
K	198±2	200±2	26400±600	26760±480
Ca	2030±10	2000±20	47000±1000	50450±550
Mn	9.73±0.09	10.0±0.1	245±8	246.3±7.1
Fe	20.1±0.9	20±1	367±10	367.5±4.3
Cu	20.5±0.9	20±1	4.69±0.15	4.70±0.14
Mo	9.8±0.1	10.0±0.1	/	/

[a]informative value

To control the accuracy of Pb determination. CRM 1573a was spiked with 1 mg/kg Pb and the analytical procedure applied. The Pb concentration determined in CRM was 0.596 mg/kg and in the spiked sample 1.582 mg/kg. The recovery calculated as the ratio between the determined concentration in the spiked sample (1.582 mg/kg) and the expected concentration (1.596 mg/kg) was 99%.

Table S4. LODs and LOQs for the determination of the elements in th enutrient solution. LODs and LOQs were calculated as the concentration providing a signal equal to 3*s* or 10*s* of the blank sample, respectively. To calculate the LODs and LOQs, 8 blank samples were analyzed by ICP-MS.

Element	LODs for total concentrations of elements in nutrient solution (μg/L)	LOQs for total concentrations of elements in nutrient solution (μg/L)
Pb	0.015	0.050
Cr	0.006	0.020
Cd	0.004	0.013
Zn	0.070	0.233

Table S5. LODs and LOQs for the determination of the elements in different parts of tomato plant (wet weight basis). LODs and LOQs were calculated as the concentration providing a signal equal to 3*s* or 10*s* of the blank sample, respectively. To calculate the LODs and LOQs, 8 blank samples were analyzed by ICP-MS.

Element	Part of the tomato plant	LODs for concentrations of elements in tomato plant (mg/kg)	LOQs for concentrations of elements in tomato plant (mg/kg)
Pb	Roots	0.00078	0.00259
	Stems	0.00021	0.00069
	Leaves	0.00035	0.00115
	Fruits	0.00009	0.00030
Cr	Roots	0.00031	0.00104
	Stems	0.00009	0.00028
	Leaves	0.00014	0.00046
	Fruits	0.00004	0.00012
Cd	Roots	0.00021	0.00069
	Stems	0.00005	0.00017
	Leaves	0.00010	0.00033
	Fruits	0.00003	0.00008
Zn	Roots	0.00363	0.01201
	Stems	0.00097	0.00324
	Leaves	0.00162	0.00540
	Fruits	0.00042	0.00140
B	Roots	1.55	5.18
	Stems	0.416	1.39
	Leaves	0.691	2.31
	Fruits	0.182	0.605
Na	Roots	0.103	0.345
	Stems	0.277	0.093

	Leaves	0.041	0.153
	Fruits	0.0121	0.0404
Mg	Roots	0.103	0.345
	Stems	0.277	0.093
	Leaves	0.041	0.153
	Fruits	0.0121	0.0404
P	Roots	0.103	0.345
	Stems	0.277	0.093
	Leaves	0.041	0.153
	Fruits	0.0121	0.0404
K	Roots	0.518	1.73
	Stems	0.139	0.463
	Leaves	0.230	0.767
	Fruits	0.061	0.202
Ca	Roots	2.59	8.63
	Stems	0.694	2.31
	Leaves	1.15	3.84
	Fruits	0.303	1.01
Mn	Roots	0.00021	0.00069
	Stems	0.00005	0.00017
	Leaves	0.00010	0.00033
	Fruits	0.00003	0.00008
Fe	Roots	0.00031	0.00104
	Stems	0.00009	0.00028
	Leaves	0.00014	0.00046
	Fruits	0.00004	0.00012
Cu	Roots	0.00021	0.00069
	Stems	0.00005	0.00017
	Leaves	0.00010	0.00033
	Fruits	0.00003	0.00008
Mo	Roots	0.00031	0.00104
	Stems	0.00009	0.00028
	Leaves	0.00014	0.00046
	Fruits	0.00004	0.00012

Table S6. The uptake of contaminants (Pb, Cr, Cd and Zn) and essential elements in the presence of contaminants at concentration levels LI and LII in different parts of the tomato. Element concentrations, determined by ICP-MS after the microwave-assisted digestion of samples, represent the average of three parallel samples and are expressed on a wet weight basis. The measurement uncertainty of ICP-MS is better than ±3, while the relative deviation in concentration between three parallel samples did not exceed ±30%.

UPTAKE OF CONTAMINANTS					UPTAKE OF ESSENTIAL ELEMENTS									
ROOTS	Pb (mg/kg)	Cr (mg/kg)	Cd (mg/kg)	Zn (mg/kg)	Mo (mg/kg)	Mn (mg/kg)	B (mg/kg)	Cu (mg/kg)	Fe (mg/kg)	Na (mg/kg)	P (mg/kg)	Mg (mg/kg)	Ca (mg/kg)	K (mg/kg)
C	0.454	0.508	0.150	11.3	1.86	2.82	7.71	1.29	43.1	438	2383	4940	6781	25938
LI	17.3	31.6	10.8	22.5	1.83	2.70	8.47	1.61	43.0	253	2149	3371	7200	25929
LII	107	332	384	551	1.77	2.92	8.81	1.75	41.2	245	2249	4672	5896	28187
STEMS	Pb (mg/kg)	Cr (mg/kg)	Cd (mg/kg)	Zn (mg/kg)	Mo (mg/kg)	Mn (mg/kg)	B (mg/kg)	Cu (mg/kg)	Fe (mg/kg)	Na (mg/kg)	P (mg/kg)	Mg (mg/kg)	Ca (mg/kg)	K (mg/kg)
C	0.006	0.018	0.009	1.17	0.241	0.320	1.92	0.188	4.20	32.8	313	1056	1409	6418
LI	0.047	0.096	0.671	7.14	0.284	0.310	1.88	0.248	6.00	24.2	326	1084	1334	6335
LII	0.187	0.227	8.50	44.6	0.504	0.400	2.43	0.427	8.50	35.1	871	1687	1760	6214
LEAVES	Pb (mg/kg)	Cr (mg/kg)	Cd (mg/kg)	Zn (mg/kg)	Mo (mg/kg)	Mn (mg/kg)	B (mg/kg)	Cu (mg/kg)	Fe (mg/kg)	Na (mg/kg)	P (mg/kg)	Mg (mg/kg)	Ca (mg/kg)	K (mg/kg)
C	0.046	0.092	0.081	4.03	1.04	2.02	9.14	0.734	24.0	31.6	695	2440	7197	5088
LI	0.073	0.209	2.87	7.45	1.27	2.09	11.1	0.801	30.5	28.1	700	2481	7158	5389
LII	0.131	0.417	21.9	26.7	2.15	1.72	14.6	1.29	43.8	39.9	868	2491	6550	6460
FRUITS	Pb (mg/kg)	Cr (mg/kg)	Cd (mg/kg)	Zn (mg/kg)	Mo (mg/kg)	Mn (mg/kg)	B (mg/kg)	Cu (mg/kg)	Fe (mg/kg)	Na (mg/kg)	P (mg/kg)	Mg (mg/kg)	Ca (mg/kg)	K (mg/kg)
C	0.0009	0.010	0.010	1.19	0.092	0.230	0.707	0.140	3.82	12.5	347	133	74.9	2635
LI	0.0011	0.028	0.248	2.17	0.102	0.132	0.697	0.238	4.60	9.36	311	138	56.5	2706
LII	0.0029	0.065	1.08	4.84	0.131	0.150	0.751	0.280	5.88	11.3	375	165	71.9	2772

C = Control, LI = Concentration level I, LII = Concentration level II

Table S7. One-Way ANOVA of Pb, Cr, Cd, and Zn concentrations in the tomato roots, stems, leaves, and fruits of control plants and plants grown in contaminated nutrient solutions at concentration levels LI and LII, with *p*-values indicating the level of significance ($p < 0.05$).

ROOTS

Pb (mg/kg wet weight)

Control	LI	LII
0,453	17,9	131
0,386	12,4	88,2
0,523	21,6	102

Anova: Single Factor

SUMMARY

Groups	Count	Sum	Average	Variance
Control	3	1,361777	0,453926	0,004664
LI	3	51,92865	17,30955	21,21289
LII	3	320,7967	106,9322	469,9465

ANOVA

Source of Variation	SS	df	MS	F	P-value	F crit
Between Groups	19653,97	2	9826,985	60,02	0,00011	5,1433
Within Groups	982,3282	6	163,7214			
Total	20636,3	8				

Cr (mg/kg wet weight)

Control	LI	LII
0,347	35,4	423
0,574	23,6	245
0,603	35,8	329

Anova: Single Factor

SUMMARY

Groups	Count	Sum	Average	Variance
Control	3	1,524048	0,508016	0,019613
LI	3	94,82466	31,60822	47,81998
LII	3	996,7587	332,2529	7920,141

ANOVA

Source of Variation	SS	df	MS	F	P-value	F crit
Between Groups	201409,1	2	100704,5	37,92	0,00039	5,1433
Within Groups	15935,96	6	2655,994			
Total	217345,1	8				

Cd (mg/kg wet weight)

Control	LI	LII
0,184	8,2	398
0,116	12,5	367
0,150	11,7	386

Anova: Single Factor

SUMMARY

Groups	Count	Sum	Average	Variance
Control	3	0,44968	0,149893	0,001163
LI	3	32,39659	10,79886	5,422661
LII	3	1151,271	383,757	250,2462

ANOVA

Source of Variation	SS	df	MS	F	P-value	F crit
Between Groups	286365,6	2	143182,8	1680	5,66295E-09	5,1433
Within Groups	511,3401	6	85,22336			
Total	286876,9	8				

Zn (mg/kg wet weight)

Control	LI	LII
11,3	18,6	549
10,4	18,4	557
12,3	30,6	546

Anova: Single Factor

SUMMARY

Groups	Count	Sum	Average	Variance
Control	3	33,98496	11,32832	0,884015
LI	3	67,63801	22,546	48,93581
LII	3	1652,505	550,8349	30,13249

ANOVA

Source of Variation	SS	df	MS	F	P-value	F crit
Between Groups	570282,4	2	285141,2	10699	2,20266E-11	5,1433
Within Groups	159,9046	6	26,65077			
Total	570442,3	8				

STEMS

Pb (mg/kg wet weight)

Control	LI	LII
0,004	0,057	0,192
0,007	0,053	0,206
0,008	0,030	0,161

Anova: Single Factor

SUMMARY

Groups	Count	Sum	Average	Variance
Control	3	0,018034	0,006011	3,27949E-06
LI	3	0,140169	0,046723	0,000216487
LII	3	0,559687	0,186562	0,000520692

ANOVA

Source of Variation	SS	df	MS	F	P-value	F crit
Between Groups	0,053811	2	0,026906	109,0	1,92133E-05	5,1433
Within Groups	0,001481	6	0,000247			
Total	0,055292	8				

Cr (mg/kg wet weight)

Control	LI	LII
0,018	0,108	0,176
0,016	0,082	0,202
0,021	0,097	0,303

Anova: Single Factor

SUMMARY

Groups	Count	Sum	Average	Variance
Control	3	0,054447	0,018149	5,42841E-06
LI	3	0,287095	0,095698	0,000175746
LII	3	0,682078	0,227359	0,004489867

ANOVA

Source of Variation	SS	df	MS	F	P-value	F crit
Between Groups	0,067118	2	0,033559	21,55	0,00182	5,1433
Within Groups	0,009342	6	0,001557			
Total	0,07646	8				

Cd (mg/kg wet weight)

Control	LI	LII
0,008	0,641	7,09
0,010	0,712	11,1
0,008	0,660	7,35

Anova: Single Factor

SUMMARY

Groups	Count	Sum	Average	Variance
Control	3	0,025505	0,008502	2,35905E-06
LI	3	2,013067	0,671022	0,001372384
LII	3	25,49034	8,496781	4,930751158

ANOVA

Source of Variation	SS	df	MS	F	P-value	F crit
Between Groups	133,7323	2	66,86616	40,67	0,00032	5,1433
Within Groups	9,864252	6	1,644042			
Total	143,5966	8				

Zn (mg/kg wet weight)

Control	LI	LII
1,19	6,42	48,6
1,17	7,87	44,8
1,16	7,14	40,2

Anova: Single Factor

SUMMARY

Groups	Count	Sum	Average	Variance
Control	3	3,519249	1,173083	0,000198369
LI	3	21,41985	7,139951	0,525157855
LII	3	133,6534	44,55113	17,70151476

ANOVA

Source of Variation	SS	df	MS	F	P-value	F crit
Between Groups	3316,855	2	1658,427	273,0	1,28471E-06	5,1433
Within Groups	36,45374	6	6,075624			
Total	3353,308	8				

LEAVES

Pb (mg/kg wet weight)

Control	LI	LII
0,032	0,079	0,148
0,058	0,079	0,102
0,047	0,061	0,144

Anova: Single Factor

SUMMARY

Groups	Count	Sum	Average	Variance
Control	3	0,136888	0,045629	0,00016
LI	3	0,218383	0,072794	0,000107
LII	3	0,394044	0,131348	0,00066

ANOVA

Source of Variation	SS	df	MS	F	P-value	F crit
Between Groups	0,011514	2	0,005757	18,63	0,00267	5,1433
Within Groups	0,001854	6	0,000309			
Total	0,013369	8				

Cr (mg/kg wet weight)

Control	LI	LII
0,080	0,178	0,508
0,109	0,169	0,312
0,087	0,279	0,431

Anova: Single Factor

SUMMARY

Groups	Count	Sum	Average	Variance
Control	3	0,275047	0,091682	0,000225
LI	3	0,626623	0,208874	0,003733
LII	3	1,251012	0,417004	0,009764

ANOVA

Source of Variation	SS	df	MS	F	P-value	F crit
Between Groups	0,162886	2	0,081443	17,80	0,00300	5,1433
Within Groups	0,027445	6	0,004574			
Total	0,190331	8				

Cd (mg/kg wet weight)

Control	LI	LII
0,065	2,74	23,1
0,103	2,89	20,0
0,074	2,99	22,5

Anova: Single Factor

SUMMARY

Groups	Count	Sum	Average	Variance
Control	3	0,242375	0,080792	0,000379
LI	3	8,615859	2,871953	0,015716
LII	3	65,64031	21,8801	2,753304

ANOVA

Source of Variation	SS	df	MS	F	P-value	F crit
Between Groups	844,3103	2	422,1551	457,3	2,76835E-07	5,1433
Within Groups	5,538798	6	0,923133			
Total	849,8491	8				

Zn (mg/kg wet weight)

Control	LI	LII
3,00	9,94	29,2
5,19	5,78	23,2
3,90	6,62	27,7

Anova: Single Factor

SUMMARY

Groups	Count	Sum	Average	Variance
Control	3	12,09025	4,030082	1,211865
LI	3	22,3356	7,445201	4,846014
LII	3	80,12105	26,70702	9,858767

ANOVA

Source of Variation	SS	df	MS	F	P-value	F crit
Between Groups	896,924	2	448,462	84,53	4,02659E-05	5,1433
Within Groups	31,83329	6	5,305549			
Total	928,7573	8				

FRUITS

Pb (mg/kg wet weight)

Control	LI	LII
0,0008	0,0010	0,0020
0,0009	0,0014	0,0034
0,0009	0,0010	0,0034

Anova: Single Factor

SUMMARY

Groups	Count	Sum	Average	Variance
Control	3	0,002579	0,00086	6,27E-09
LI	3	0,003437	0,001146	6,19E-08
LII	3	0,008781	0,002927	6,38E-07

ANOVA

Source of Variation	SS	df	MS	F	P-value	F crit
Between Groups	7,53E-06	2	3,76E-06	15,99	0,00394	5,1433
Within Groups	1,41E-06	6	2,35E-07			
Total	8,94E-06	8				

Cr (mg/kg wet weight)

Control	LI	LII
0,012	0,020	0,065
0,011	0,030	0,072
0,009	0,033	0,058

Anova: Single Factor

SUMMARY

Groups	Count	Sum	Average	Variance
Control	3	0,031344	0,010448	2,74E-06
LI	3	0,082749	0,027583	4,58E-05
LII	3	0,195939	0,065313	5,15E-05

ANOVA

Source of Variation	SS	df	MS	F	P-value	F crit
Between Groups	0,004727	2	0,002364	70,86	6,70089E-05	5,1433
Within Groups	0,000200	6	3,34E-05			
Total	0,004927	8				

Cd (mg/kg wet weight)

Control	LI	LII
0,012	0,253	1,06
0,010	0,218	1,22
0,008	0,276	0,951

Anova: Single Factor

SUMMARY

Groups	Count	Sum	Average	Variance
Control	3	0,029979	0,009993	3,97E-06
LI	3	0,746176	0,248725	0,000862
LII	3	3,231741	1,077247	0,017828

ANOVA

Source of Variation	SS	df	MS	F	P-value	F crit
Between Groups	1,882473	2	0,941236	151,1	7,38544E-06	5,1433
Within Groups	0,037388	6	0,006231			
Total	1,919861	8				

Zn (mg/kg wet weight)

Control	LI	LII
1,27	2,49	4,83
1,09	1,94	5,00
1,19	2,08	4,69

Anova: Single Factor

SUMMARY

Groups	Count	Sum	Average	Variance
Control	3	3,555794	1,185265	0,008369
LI	3	6,514334	2,171445	0,081338
LII	3	14,51603	4,838675	0,024369

ANOVA

Source of Variation	SS	df	MS	F	P-value	F crit
Between Groups	21,43408	2	10,71704	281,8	1,16833E-06	5,1433
Within Groups	0,228153	6	0,038025			
Total	21,66223	8				

Table S8. Estimated daily intake and hazard quotient for toddler and adult population groups.

Adults					
Concentration level		LI		LII	
Exposure		Average	High	Average	High
Estimated daily intake (mg/kg bw day)	Cd	0.0001	0.0004	0.0005	0.0021
	Pb	0.0000	0.0000	0.0000	0.0000
HQ	Cd	0.27	**1.17**	**1.32**	**5.76**
	Pb	0.0001	0.0003	0.0005	0.0016

Children (1-3 years)					
Concentration level		LI		LII	
Exposure		Average	High	Average	High
Estimated daily intake (mg/kg bw day)	Cd	0.0002	0.0008	0.0011	0.0050
	Pb	0.0000	0.0000	0.0000	0.0000
HQ	Cd	0.53	**2.30**	**3.22**	**14.02**
	Pb	0.0002	0.0006	0.0012	0.0039

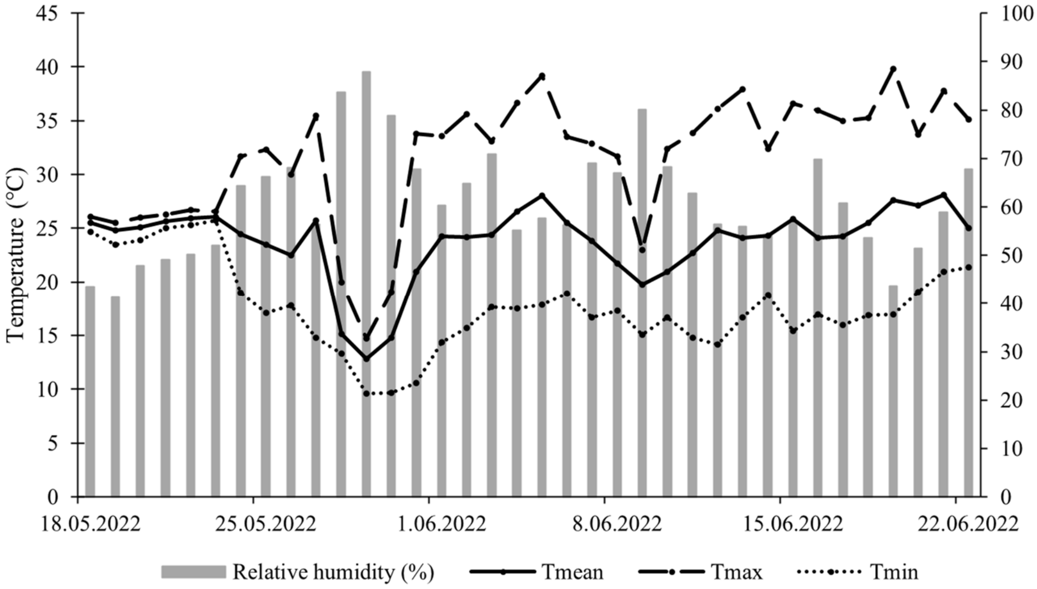

Figure S1. Daily mean air temperature (T_{mean}) and relative humidity (RH) during the plant growth period in Ljubljana, 2022. T_{mean} ranged from 13 to 28 °C, with recorded extremes of 10 °C (T_{min}) and 40 °C (T_{max}).

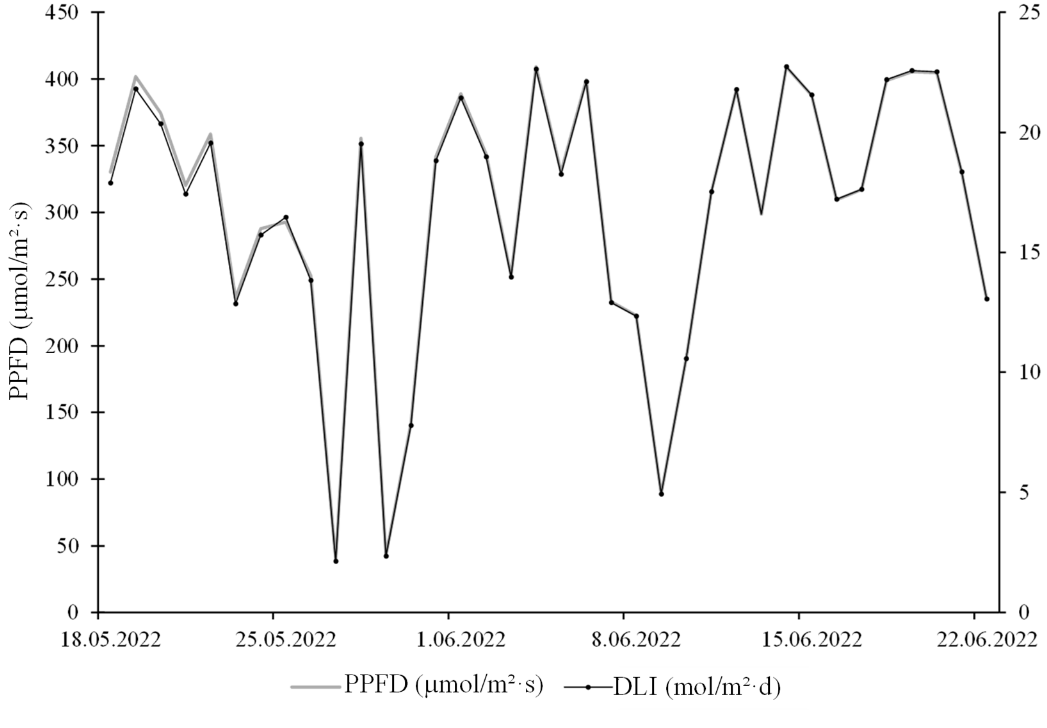

Figure S2. Photosynthetic Photon Flux Density (PPFD, primary axis) and Daily Light Integral (DLI, secondary axis) during the growth period in Ljubljana, 2022.
